# IgG Subtype Response against Virulence-Associated Protein A in Foals Naturally Infected with *Rhodococcus equi*

**DOI:** 10.3390/vetsci11090422

**Published:** 2024-09-09

**Authors:** Yuya Mizuguchi, Nao Tsuzuki, Marina Dee Ebana, Yasunori Suzuki, Tsutomu Kakuda

**Affiliations:** 1 Mitsuishi Animal Medical Center, Hokkaido 059-3105, Japan; yuuya_mizuguchi_p1@nosai-do.or.jp; 2 Department of Veterinary Medicine, Rakuno Gakuen University, Hokkaido 069-8501, Japan; n-tsuzuki@rakuno.ac.jp; 3 Laboratory of Animal Hygiene, Faculty of Veterinary Medicine, School of Veterinary Medicine, Kitasato University, Aomori 034-8628, Japan; vm19022@st.kitasato-u.ac.jp (M.D.E.); ysuzuki@vmas.kitasato-u.ac.jp (Y.S.)

**Keywords:** *Rhodococcus equi*, prognosis, IgG subtype, polarization of Th-cell response

## Abstract

**Simple Summary:**

*Rhodococcus equi* infection causes life-threatening bacterial pneumonia in foals, resulting in significant economic losses to equine farms. Due to the lack of an effective vaccine, infected foals receive intensive antibiotic treatment. However, the emergence of antibiotic-resistant bacteria in recent years has become problematic. Thus, appropriate use of antibiotics is required. Horses that die from *R. equi* infection tend to show specific patterns of IgG subtype responses. Therefore, this study focused on qualitative differences in antibody responses to *R. equi* between resistant and susceptible foals as a means of predicting *R. equi* susceptibility. These findings may be useful in predicting which foals require treatment.

**Abstract:**

*Rhodococcus equi* is an intracellular bacterium that causes suppurative pneumonia in foals. T-helper (Th) 1 cells play an important role in the protective response against *R. equi*. In mice and humans, the directionality of IgG switching reflects the polarization of Th-cell responses, but this has not been fully elucidated in horses. In this 4-year study, we classified *R. equi*-infected foals into surviving and non-surviving group and investigated differences in IgG subclass response to virulence-associated protein A, the main virulence factor of *R. equi*, between the groups. IgGa, IgGb, and IgG(T) titers were significantly higher in the non-surviving group compared with the surviving group. The titers of IgGa and IgG(T), IgGb and IgG(T), and IgGa and IgGb, respectively, were positively correlated, and the IgG(T)/IgGb ratio in the non-surviving group was significantly higher than that in the surviving group. The IgG(T) titer tended to increase more than the IgGa and IgGb titers in the non-surviving group compared with the surviving group. Our findings suggest that the IgG(T) bias in IgG subclass responses reflects the immune status, which exacerbates *R. equi* infection.

## 1. Introduction

*Rhodococcus equi* (*R. equi*) causes suppurative pneumonia in foals aged 1–5 months, leading to severe economic losses in horse-farming areas worldwide each year [1,2,3]. *R. equi* is widely distributed in soil, and the rate of infection is high on farms with high levels of contamination [4]. Because there is no effective vaccine, clinically affected foals are diagnosed early and treated with antibiotics [5].

*R. equi* is a Gram-positive intracellular bacterium that survives and multiplies within the phagosomes of macrophages [6]. It harbors a virulence plasmid that encodes for virulence-associated protein A (VapA), which is secreted within the macrophage phagosome to prevent decreased intravesicular pH, facilitating *R. equi* survival [7,8]. Although humoral immune responses have been reported to induce protective effects by producing specific antibodies, cellular immune responses are considered of primary importance for bacterial elimination [3]. In a mouse model in which the immune response to *R. equi* has been well studied, transfer of a T-helper (Th) 1 cell line producing interferon gamma (IFN-γ) promoted clearance of *R. equi* from the lungs, whereas transfer of a Th2 cell line producing interleukin (IL)-4 failed to protect recipient mice against *R. equi* infection [9,10]. It has been speculated that the production of *R. equi*-specific cytotoxic T lymphocytes and IFN-γ are involved in *R. equi* elimination in horses [11,12,13,14,15], but this has yet to be confirmed.

B cell isotype switching is regulated by the types of cytokines produced by T follicular helper (Tfh) cells [16]. Depending on the nature of immune stimulation, Tfh cells polarize in parallel with Th1/Th2 polarization [17]. Intracellular microbial infections, such as *Leishmania* and *Mycobacteria*, induce Th1-type cellular responses [18,19,20], and helminth infections induce Th2-type cellular responses [21]. The Tfh subset that produces IFN-γ induces differentiation of B lymphocytes that produce IgG2a in secondary lymphoid tissues, while the Tfh subset that produces IL-4 and IL-13 induces differentiation of B lymphocytes that produce IgE, IgG1 (mouse), and IgG4 (human) [22]. Therefore, the IgG subtype produced in response to infection reflects the bias of the Th-cell response directed toward the pathogen.

In horses, Protective immunity to equine herpesvirus type 1 infection was characterized by a polarized IFN-γ response and a predominance of IgGa and IgGb [23]. In contrast, increased concentration of IgG(T) was observed in horses infected with intestinal nematodes such as *Anoplacephala perfoliata* and *Strongylus vulgaris*, which induce Th2 response characterized by high levels of IL-4 [24]. In foals experimentally infected with *R. equi*, it has been reported that serum IgGa and IgGb levels positively correlated with IFN-γ mRNA levels and that IgGc and IgG(T) levels show a positive correlation with IL-4 mRNA level, when mononuclear cells isolated from bronchial lymph nodes of *R. equi*-infected foals were stimulated with *R. equi* antigen [25].

IgGb (IgG4 and IgG7 according to new terminology) is the most prevalent Ig subtype in adult equine serum, followed by IgG(T) (IgG3 and IgG5), IgGa (IgG1), and IgGc (IgG6) [26]. A significant increase in serum IgGa, IgGb, and IgGc against *R. equi* was reported in foals experimentally infected with *R. equi* [25], and VapA-specific IgG(T) levels have been reported to increase over time in naturally infected foals [27]. However, information regarding the relationship between disease progression and the profile of the IgG subtypes produced is lacking. Therefore, this 4-year study analyzed the relationship between *R. equi* infection and IgG subtype production by measuring the serum levels of VapA-specific IgG subtypes in foals naturally infected with *R. equi* and comparing the IgG subtype responses between the foals that died and the foals that survived after receiving treatment.

## 2. Materials and Methods

### 2.1. Serum Sample Collection

We collected serum from 43 thoroughbred foals born on horse farms in the Hidaka region, Hokkaido, Japan, during 2019–2022. All farms had a history of recurrent pneumonia in foals caused by *R. equi*. *R. equi* infection was confirmed by isolation from tracheobronchial aspirates and/or by thoracic ultrasonography confirmation of abscess formation in addition to the manifestation of clinical signs of bronchopneumonia caused by *R. equi*. Following diagnosis, foals were treated with a combination of antibiotics, such as a macrolide and rifampicin. Of the 43 foals in our study cohort, 6 died and 37 survived. The blood samples were collected at the first visit and at follow-up examinations 4–10 days apart. If the sick foals recovered, sampling was discontinued. The number of blood samples collected from each foal varied, with four samples taken from 1 (2%) foal, three from 3 (7%) foals, two from 20 (47%) foals, and one from 19 (44%) foals. The collected blood was centrifuged (2000× *g*, 10 min), and the resultant serum samples were cryopreserved at −30 °C.

### 2.2. Control Serum

The serum collected from an adult mare that had been vaccinated twice with RHODOVAC (PRO-SER S.A, Buenos Aires, Argentina) was kindly gifted from Equine Research Institute in Japan Racing Association. The serum was aliquoted and cryopreserved at −30 °C.

### 2.3. Recombinant GST-VapA

Recombinant GST-VapA was expressed and purified as described previously [28]. Briefly, plasmid pGEX-4T-1 (GE Healthcare Life Sciences, USA) was used to express *R. equi* VapA as a GST fusion protein in *E. coli* BL21 cells that had been cultured in Lauria–Bertini broth supplemented with ampicillin (50 μg/mL) at 37 °C. GST-VapA expression was induced by the addition of 1 mM IPTG at a final concentration for 5 h at 30 °C. Bacteria were harvested and disrupted by sonication, and GST-VapA was purified using affinity chromatography with glutathione sepharose 4B resin (GE Healthcare Life Sciences, Chicago, IL, USA).

### 2.4. Enzyme-Linked Immunosorbent Assay (ELISA)

We coated 96-well microplates with recombinant GST-VapA (1 µg/well) in carbonate buffer. Following overnight incubation at 4 °C, a blocking buffer containing block ACE (20% [*v*/*v*] in phosphate-buffered saline [PBS]) (KAC, Hyogo, Japan) was added. The serum samples were diluted in PBS with 0.05% Tween-20 (PBST), added to duplicate wells, and incubated at 37 °C for 1 h. Then, any of goat anti-horse IgG antibody(Ab) conjugated with horseradish peroxidase (HRP), murine anti-horse IgGa monoclonal Ab (Bio-Rad, Hercules, CA, USA), anti-IgGb monoclonal Ab (Bio-Rad), and goat anti-IgG(T) polyclonal Ab-HRP (Bio-Rad, USA) were added and incubated at 37 °C for 1 h. The IgGa and IgGb plates were washed, and goat anti-mouse IgG-HRP was added and incubated at 37 °C for 30 min. The plates were washed with PBST between each step. Following substrate addition and incubation for 10 min at 37 °C, absorbance was read at 490 nm using a plate reader. The absorbance values were converted to ELISA units utilizing a cubic polynomial formula from the standard curve generated for Igs and each IgG subclass using the control serum. The concentration of Igs and each IgG subclass when the control serum was diluted 1024-fold (Ig), 128-fold (IgGa), 256-fold (IgGb), and 512-fold (IgG(T)), was set as 1 unit, respectively.

### 2.5. Data Analysis

Two-tailed Student *t*-test was used to compare ELISA units or ratios between the surviving and dead groups. *p*-values < 0.05 were considered statistically significant. The strength of the relationship between two IgG subclasses was determined using Pearson product-moment correlation.

## 3. Results

We initially planned to group the foals on the basis of clinical symptom severity. However, in clinical practice, antibiotic treatments mask susceptibility to *R. equi*, so differentiation between foals that spontaneously recovered and foals whose clinical symptoms would have worsened without treatment was impossible. For more appropriate grouping, we hypothesized that foals that died despite treatment would have different responses to *R. equi* compared with foals that survived. Thus, we grouped the foals into a non-surviving group and a surviving group.

**Figure 1:**
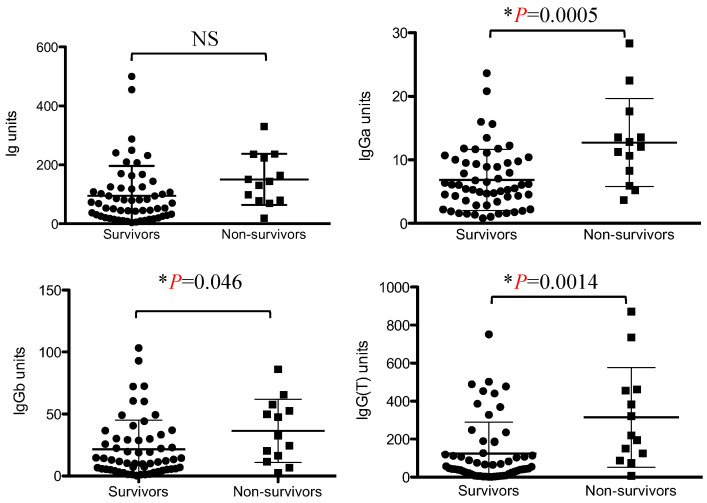
Comparison of the Ig, IgGa, IgGb, and IgG(T) titers between the non-surviving and surviving groups. Values are expressed as ELISA units. Asterisks indicate statistically significant differences (*p* < 0.05). NS, not significant.

The total Immunoglobulin titer against VapA tended to be higher in the dead group than in the surviving group, but the difference was not statistically significant. Regarding the IgG subclasses, IgGa, IgGb, and IgG(T) titers were significantly higher in the non-surviving group than in the surviving group (Figure 1).

**Figure 2:**
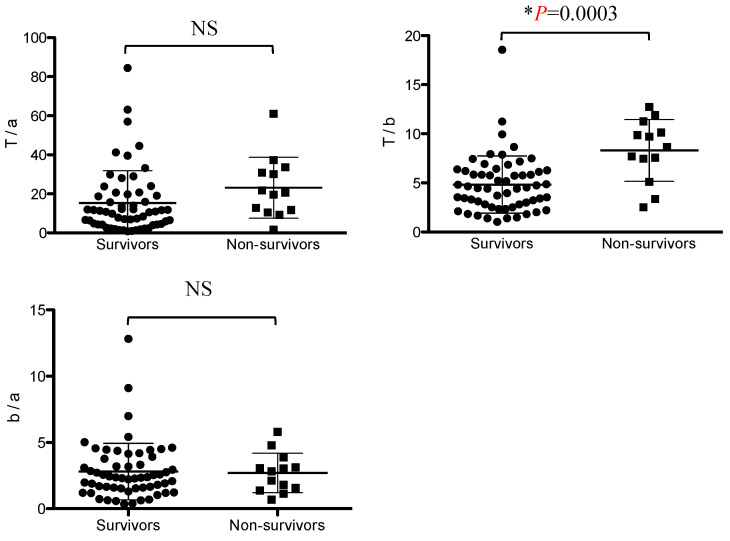
Comparison of ratios of IgG(T)/IgGa, IgG(T)/IgGb, and IgGb/IgGa in the non-surviving and surviving groups. Asterisks indicate statistically significant differences (*p* < 0.05). NS, not significant.

The IgG subclass ratio in mice and humans reflects Th1/Th2 polarity. A comparison of IgG subclass ratios between the surviving group and the non-surviving group revealed that the IgG(T)/IgGb (T/b) ratio was significantly higher in the non-surviving group than in the surviving group. The IgG(T)/IgGa ratio tended to be higher in the non-surviving group than in the surviving group, but the difference was not significant. There was no significant difference in the IgGb/IgGa ratio between the non-surviving and surviving groups (Figure 2).

**Figure 3:**
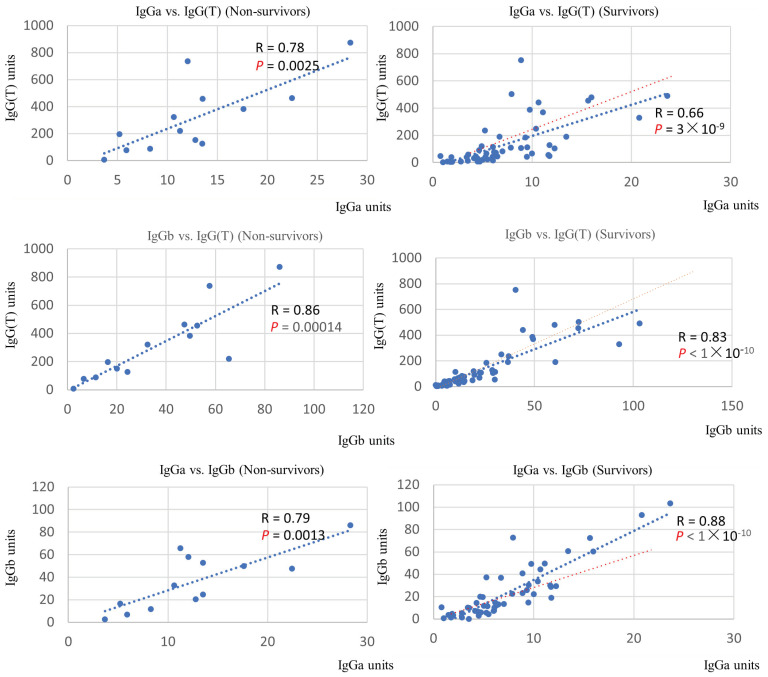
Correlation between serum levels of IgG subtypes in the non-surviving and surviving groups. The regression line (blue dots) and multiple correlation coefficient (R) are indicated. Results of tests of no correlation are expressed as *p*-values. Red dots indicate the regression line of the non-surviving group for each IgG subtype.

Examination of the quantitative correlation between the different IgG subclasses revealed a positive correlation for all combinations. Comparison of the slope of the regression line between the non-surviving and surviving groups revealed that the association between IgGb and IgG(T) was the most different (Figure 3).

We investigated whether the T/b ratio could be useful for predicting prognosis in *R. equi* infection. We tentatively set a cutoff value of 7.45 to maximize the sum of sensitivity and specificity [29] and examined whether the T/b ratio could be used for distinguishing between the sera of non-surviving and surviving foals. Our findings revealed that the T/b ratio had a sensitivity and specificity of 77% and 88%, respectively, for distinguishing between the non-surviving and surviving groups. Of the six fatal cases, three had a T/b ratio > 7.45 at the first visit and two had a T/b ratio > 7.45 within 1 week after the first visit (Table 1).

## 4. Discussion

The introduction of lung ultrasonography in the diagnosis of *R. equi* revealed that up to 80% of foals in farms where *R. equi* is endemic become infected with *R. equi* and develop lung abscesses. Although the majority of cases resolve spontaneously without treatment, 10–20% of foals with lesions show clinical symptoms, leading to death in severe cases [25,30]. Because there are no reliable clinical diagnostic markers capable of predicting prognosis, foals susceptible to *R. equi* cannot be targeted for treatment. Our study revealed significant differences in IgG subclass titers between the non-surviving and surviving groups. However, because there was considerable overlap in the range of titers between the groups, we considered that the use of IgG subclass titers as prognostic markers would be difficult. In contrast, the T/b ratio was capable of distinguishing between samples from the non-surviving and surviving groups, with a sensitivity of 77% and a specificity of 88%. This suggests that the T/b ratio batter reflects the immune status of the foals with worsening infection than the IgG subclass titers.

*R. equi* does not induce disease in immunocompetent adult horses, and adult horses were reported to effectively clear an intrabronchial challenge with a virulent strain of *R. equi* [31]. Furthermore, the increase in IgG subclasses against VapA was reported as more pronounced for IgGb and IgGa than for IgG(T) in adult horses experimentally infected with *R. equi* [31]. In contrast, IgGa and IgG(T) titers increased in foals after challenge infection with *R. equi*, and IgG(T) increased over time in cases of natural infection of foals with *R. equi* [27]. Therefore, IgGa and IgGb production is thought to be related to the resistance of adult horses against *R. equi*, and IgG(T) production is thought to be related to the susceptibility of the foals to *R. equi*. In our study, IgGa, IgGb, and IgG(T) Ab responses were significantly higher in the non-surviving group compared with the surviving group. The production of IgG subclasses has been reported to exhibit polarization in mice. This phenomenon is typically explained by classifying T cells into two distinct subsets, Th1 and Th2 [19]. However, some have argued that this model may be overly simplistic [32,33]. An alternative model suggests that cytokine production by T cells should be viewed as a continuous spectrum [32]. In this study, a quantitative correlation between IgG subclasses was examined, revealing positive correlations across all combinations in both the surviving and non-surviving groups. This finding suggests that polarization of IgG subclasses is not clearly observed in foals infected with *R. equi*. Nevertheless, it is evident that the production of each IgG subclass reflects a complex immune response. In the non-surviving group, the increase in IgG(T) tended to exceed that of IgGa and IgGb. Therefore, it is suggested that foals with worsening infection experience a cytokine environment that induces a stronger overall IgG(T) bias (Th2 bias), whereas foals showing resistance to infection exhibit a cytokine environment that diminishes the IgG(T) bias by enhanced production of Th1 cytokines.

Experimental infections of foals with low doses of virulent *R. equi* have been reported to cause subclinical infections and increased IgGa, IgGb, and IgGc titers [34]. In contrast, higher doses resulted in severe lung lesions, clinical symptoms, and a high IgG(T) titer, although it was unclear whether Th2 bias was the cause of severe disease. If Th2 bias is responsible for infection severity, then such foals may show Th2 bias relatively early in the infection. In our study, 3/6 (50%) dead foals had a T/b ratio > 7.45 at the first visit. Furthermore, the T/b ratio of two other foals in the non-surviving group increased from 2.5 and 5.1 to 10.8 (4 days after the first visit) and 9.1 (2 days after the first visit), respectively. However, a limitation of our study is that the number of non-surviving foals in our cohort was low, and this may not be an accurate reflection of the overall population. Thus, further studies on larger populations are necessary to confirm the usefulness of the T/b ratio for identifying foals susceptible to *R. equi* infections and subsequently minimizing antibiotic use.

The emergence of drug-resistant *R. equi* is problematic and can be minimized by reducing inappropriate antibiotic treatments [35,36]. This can only be achieved by distinguishing between the foals that do require antibiotic treatment for *R. equi* infection and those that do not require treatment at the early stage of infection.

## 5. Conclusions

Foals infected with *R. equi* produce VapA-specific IgGa, IgGb, and IgG(T) antibodies, and their levels are positively correlated. However, IgG(T) bias is observed in foals with worsening infections. This may be applied as a predictor of prognosis.

## Figures and Tables

**Table 1:** Number of non-surviving and surviving foals with a T/b ratio above or below 7.45.

T/B Ratio	Non-Survivors	Survivors	Sum
>7.45	10	7	17
≤7.45	3	51	54
Sum	13	58	71

## Data Availability

The data presented in this study are available on request from the corresponding author.
